# Abused Children Experience High Anger Exposure

**DOI:** 10.3389/fpsyg.2019.00440

**Published:** 2019-03-05

**Authors:** Rista C. Plate, Zachary Bloomberg, Daniel M. Bolt, Anna M. Bechner, Barbara J. Roeber, Seth D. Pollak

**Affiliations:** ^1^Department of Psychology, University of Wisconsin–Madison, Madison, WI, United States; ^2^Waisman Center, University of Wisconsin–Madison, Madison, WI, United States

**Keywords:** child maltreatment, anger, physical abuse, parents, children

## Abstract

Childhood maltreatment is a critical problem in the United States. Much attention has been paid to the negative outcomes suffered by victims of abuse. Less attention has been devoted to understanding the emotional environments of maltreated children. One assumption, which has stood without empirical test, is that abused children encounter a high degree of anger in their home environments. Anger exposure is thought to be a source of stress for children in abusive environments and a potential link between the experience of abuse and the development of health and behavioral problems. We tested this notion by assessing data on over 1,000 parents and guardians of 3- to 17-year-old children who were participants in child development studies. Abuse was measured via records from Child Protective Services regarding substantiated and unsubstantiated claims of abuse as well as parent/guardian report. We compared self-reported experiences of anger from parents/guardians of children who have experienced abuse with those who have not. We found support for the claim that caregivers of abused children experience and express high levels of anger. Better characterization of the emotional environments in which abused children develop is critical for understanding how and why abuse affects children and has important implications for informing interventions.

## Introduction

Child maltreatment is a significant public health concern in the United States. In 2016, 676,000 children were found to be substantiated victims of abuse or neglect (U.S. Department of Health & Human Services et al., 2018). Despite attention and efforts to reduce childhood abuse (Reynolds and Robertson, 2003), the number of victims continues to increase. Moreover, the instance of childhood abuse is likely higher than recorded, given that many cases are not substantiated or reported.

Victims of abuse are at risk for myriad poor physical and mental health outcomes (McLaughlin, 2016). For several decades, anger in the home environment has played a central role in theories about how and why abused children develop health and behavioral problems. Given that abuse is hostile or aggressive by nature, *the assumption* that abusive caregivers express – and that abused children witness – a lot of anger is reasonable. Many of the major theoretical accounts about the etiology of problems linked to abuse take high levels of anger exposure as an *a priori* assumption. For example, empirical studies drawn from a variety of foci such as social learning (Larrance and Twentyman, 1983; Bousha and Twentyman, 1984), perceptual learning (Pollak et al., 1997, 2001), emotion socialization (Perlman et al., 2008), attachment (Dozier et al., 2008), trauma (Briggs-Gowan et al., 2012, 2019), and gene × environment effects (Jaffee and Price, 2007) imply that their results are due, in part, to abused children’s exposure to high levels of anger. For example, that physically abused children discriminate between emotions differently than neglected or non-maltreated children is thought to result from exposure to an environment rich in emotional threat cues (Pollak et al., 2000). Even accounts that focus directly on specific aspects of abusive parenting – such as deficits in self-control and problem-solving skills – had to assume high levels of familial anger (Azar et al., 1984; Cowell et al., 2015). In addition, anger expression has been correlated with child abuse potential (Rodriguez and Green, 1997; Rodriguez, 2010).

Here, we sought to answer one fundamental question: Is there evidence to support the claim that children who are developing in abusive families are exposed to more frequent and/or more severe expressions of anger? To address this question, we collected data about anger expression from parents and guardians whose children participated in a variety of child development studies.

## Materials and Methods

### Participants

Participants were 1019 parents and guardians of children who visited our laboratory to participate in one of 39 child development studies from 2000 to 2017 (see Supplementary Materials for participant recruitment and exclusions). Parents and children gave informed, written consent for the study in which they participated (children under age 11 years old provided informed, verbal assent). Participants were mothers (87%), fathers (11%), and legal guardians (or unreported, 2%). Parents and guardians were between 23 and 73 years of age (*M* = 41.12, *SD* = 7.39, 79 did not report age) and their children were between 3 and 17 years of age (*M* = 9.69, *SD* = 2.87, 47% female) when the data were collected.

#### Determination of Abuse Status

We defined abuse as substantiated reports with Child Protective Services (CPS) and/or scores greater than or equal to 20 on the physical assault portion of a commonly used scale to assess childhood maltreatment (Conflict Tactics Scale Parent-Child Version; CTSPC; Straus et al., 1998; see Supplementary Materials for sample items). Parents/guardians of 106 children were included in the Abuse group based on high CTSPC and/or CPS reports.

We included parents/guardians in the No Abuse group if they scored less than 10 on the CTSPC and had no CPS records. Two hundred six parents/guardians had unsubstantiated or on-going reports of child maltreatment with the county, notations of “concern” without sufficient evidence from CPS, elevated CTSPC scores but fell short of the threshold (i.e., scores that were >10 but <20), and/or another child with substantiated maltreatment. We decided to include these participants in the Abuse group. Based on our experience and the suspected underreporting of child abuse, we reasoned these are cases where experts have good reason to be concerned about child welfare, but not yet sufficient evidence to trigger social services involvement. Note that if this assumption is incorrect, we have merely added noise to our data, dampening the effect of interest. The final groups of interest included 312 parents/guardians of children who have likely experienced abuse (Abuse group) and 707 parents/guardians of children who have no indications of having experienced abuse (No Abuse group).

### Measure

The *State-Trait Anger Expression Inventory Revised Second Edition* (STAXI-2; Spielberger and Sydeman, 1994) was used to measure participants’ experience, expression, and control of anger. Fifty-seven items in six subscales and one index were rated on a 4-point forced-choice response scale (see Supplementary Materials for subscale, reliability, and validity information). The 32-item Anger Expression Index (AX-Index) provides an overall index of anger expression, with scores ranging from 0 to 96.

## Results

### Comparison of Group Demographics

The Abuse and No Abuse groups did not differ in age of the child participating in the study, the child’s sex, or proportion of mothers versus not mothers (i.e., fathers, legal guardians; means and comparisons presented in Table 1). Parents/guardians of the Abuse group were younger than those in the No Abuse group and had lower SES than those in the No Abuse group (Hollingshead Four-Factor Index of Socioeconomic Status; Hollingshead, 1975; 234 participants did not report SES).

**Table 1:** Participant and child demographics by group.

	Abuse	No Abuse	Comparison
Child age [Mean (SD)]	9.54 (2.77)	9.76 (2.92)	*b* = -0.221, *F*(1,1017) = 1.286, *p* = 0.257, *R*^2^ = 0.001
Child sex (N)	145 female, 167 male	334 female, 373 male	*X*^2^(1) = 0.025, *p* = 0.874
Respondent (N)	266 mothers, 46 not mothers	619 mothers, 88 not mothers	*X*^2^(1) = 0.809, *p* = 0.369
Parent/guardian age [Mean (SD)]	39.45 (8.02)	41.81 (7.01)	*b* = -2.359, *F*(1,938) = 20.17, *p* < 0.001, *R*^2^ = 0.021
SES [Mean (SD)]	40.45 (15.6)	51.74 (11.35)	*b* = -11.284, *F*(1,783) = 126.2, *p* < 0.001, *R*^2^ = 0.139
Child race (N)		
Black	101	66
White	138	502
Asian	14	39
Hispanic	10	9
Multi-racial	33	51
Other/not-reported	16	39


### Do Abused Children Experience High Anger Exposure?

We expected a linear relationship between group and anger exposure, therefore we regressed AX-Index on group (Abuse = 0.5, No Abuse = -0.5). Parents/guardians of abused children had higher anger expression scores compared to parents/guardians of not abused children [*b* = 4.462, *F*(1,1017) = 54.73, *p* < 0.001, *R*^2^ = 0.05]. We separately compared children with unsubstantiated reports of child abuse against the No Abuse group. Parents/guardians of children with unsubstantiated abuse also reported higher anger expression compared to parents/guardians of not abused children [*b* = 2.948, *F*(1,911) = 18.21, *p* < 0.001, *R*^2^ = 0.02]; however, caregivers of children with unsubstantiated abuse reported lower anger expression than those of abused children [*b* = 4.45, *F*(1,310) = 15.88, *p* < 0.001, *R*^2^ = 0.05]. Therefore, there may be meaningful variation based on amount of abuse. Consistent with this idea CTSPC scores were correlated with anger expression; parents/guardians who reported higher rates of child abuse reported greater anger expression, *r*(1005) = 0.191, *p* < 0.001, 95% CI (0.131, 0.250).

Because the groups differed by parent/guardian age and SES, we ran a second model including these variables to ensure that the effect was not being driven by alternative factors. We regressed anger expression on group, respondent age (mean-centered), SES (mean-centered), and their interaction terms (Note: 251 observations did not include respondent age or SES and were therefore excluded from the analysis including covariates). Controlling for parent/guardian age and SES, the effect of group on anger expression maintained significance, *b* = 3.382, *t*(760) = 4.292, *p* < 0.001, Δ*R*^2^ = 0.023. Additionally, there was a significant effect of SES; as SES increased, reported anger expression decreased, *b* = -0.063, *t*(760) = -2.415, *p* = 0.016, Δ*R*^2^ = 0.007. However, the effect of SES explained less variance in the model than the effect of group. No other effects or interactions in the model were significant (*p*s > 0.580). While we were primarily interested in overall anger expression, subscale comparisons are presented in Table 2.

**Table 2:** Sample means (standard deviations) for STAXI measures and group comparisons.

Measure	Description	Abuse Mean (SD)	No Abuse Mean (SD)	Comparison
AX_Index	Anger expression	53.47 (9.57)	49.01 (8.55)	*F*(1,1017) = 54.73, *p* < 0.001, *R*^2^ = 0.05
S_Ang	State anger	47.49 (8.06)	45.01 (3.51)	*F*(1,1012) = 46.67, *p* < 0.001, *R*^2^ = 0.044
T_Ang	Trait anger	46.72 (10.24)	43.09 (7.71)	*F*(1,1017) = 38.74, *p* < 0.001, *R*^2^ = 0.038
AX-I	Anger expression-inward	49.91 (10.64)	47.04 (9.29)	*F*(1,989) = 18.40, *p* < 0.001, *R*^2^ = 0.018
AX-O	Anger expression-outward	52.31 (11.29)	48.24 (9.1)	*F*(1,1016) = 37.09, *p* < 0.001, *R*^2^ = 0.035
AC-I	Anger control-inward	46.67 (9.38)	49.49 (9)	*F*(1,1017) = 20.69, *p* < 0.001, *R*^2^ = 0.020
AC-O	Anger control-outward	45.8 (10.76)	48.98 (10.13)	*F*(1,1016) = 20.52, *p* < 0.001, *R*^2^ = 0.020


*All effects maintained statistical significance (*p*s ≤ 0.01) when respondent age and SES were included in the model*.

## Discussion

We set out to test whether abused children encounter more expressed anger in their family environments, and we found that parents/guardians of abused children do indeed report greater anger expression than those of not abused children. This greater amount of anger spans state and trait anger, and parents/guardians of abused children report less ability to control their expression of anger.

While we did not initially predict a relation between anger expression and SES; we discovered this relation by including SES in the model. However, the relation is not entirely surprising. Individuals with fewer financial and educational resources likely encounter greater barriers than those with more resources. Such increased stress could influence the amount of anger an individual experiences, as well as the individual’s ability to control its expression. Further, this is not the first investigation to note concurrence between abuse and income. Both physically abused children and children from very low-income families have shown neurocognitive delays and behavioral problems (Hanson et al., 2013). Alternatively, families with higher levels of education may pay greater attention to societal expectations, and therefore may simply be better at tempering how they complete questionnaires in the laboratory environment.

### Implications of Exposure to Anger

Exposure to high levels of hostility in the home environment may contribute to downstream effects of important mechanisms linking maltreatment to adverse outcomes. For example, exposure to consistent anger, signaling a threatening environment, may influence children’s diurnal cortisol rhythms, which moderate the relation between abuse and aggressive behavior (Bernard et al., 2015). Additionally, the amount of anger a child observes influences how children form emotion category representations, which in turn influence the judgments that people make about others (Etcoff and Magee, 1992; Campanella et al., 2002; Plate et al., 2018). Consistent with this idea, abused children perceptually discriminate emotional faces differently from non-abused children (Pollak and Kistler, 2002). Interpreting emotions is a key component of social competence, and failure to master this critical skill could have damaging effects on children’s social development.

Another implication of greater anger exposure might be children’s observational learning. Research has long shown that a primary source of children’s learning occurs through observations of others (e.g., Bandura and Walters, 1977). When one of a child’s primary models expresses great amounts of anger, the child may adopt those standards of expression. Further, these data suggest that parents/guardians of maltreated children demonstrate less ability to control their anger (as evidenced by differences on anger control subscales), therefore limiting the observational opportunities for these children to learn emotion regulation, a skill often reduced in maltreated children (Heleniak et al., 2016).

### Limitations

One limitation is that anger expression was determined via self-report alone. It is unlikely that parents exaggerated their anger expression. However, it is possible that respondents underestimated or inaccurately reported anger expression. This could happen intentionally, as respondents modulate their responses according to suspected norms, or because individuals may not have insight into their own emotional lives. Additionally, because CPS reports vary greatly in the level of detail available, there were some cases where we could not ascertain if the respondent was deemed responsible for instances of substantiated abuse. This uncertainty potentially works against the effects we were testing, but should still be considered in interpreting the results. Nevertheless, what parents are choosing to reveal suggests that abused children not only experience direct acts of aggression, but abused children likely develop within hostile family contexts.

Another limitation is that many children experience more than one type of maltreatment. Here, we focused on abuse, specifically physical abuse as measured by the CTSPC. However, there is a high co-occurrence amongst different types of maltreatment (Vachon et al., 2015), and certain types of abuse (i.e., emotional abuse) can be particularly difficult to detect or substantiate. Additionally, the effect size for the relation between abuse and anger exposure is small. It is likely that myriad other factors also contribute to the complex experiences of children living in abusive environments.

## Conclusion

We addressed one key question, namely, whether abused children encounter a lot of anger in their home environment. While seemingly simple, this empirical question has been taken as an assumption in the literature. Here we provide evidence that abused children are exposed to greater amounts of caregiver anger than children who have not been abused. Questions remain regarding the implications of this finding for mechanisms linking maltreatment to adverse outcomes. Given the persistent frequency of childhood maltreatment, as well as the detrimental and expansive consequences, these questions remain critical to address in order to advance scientific understanding and policy to protect vulnerable youth.

## Data Availability

The de-identified dataset and analysis script are available at: https://osf.io/35xzt/.

## Ethics Statement

The protocol was approved by the University of Wisconsin Institutional Review Board. Written informed consent was obtained from all adult participants and from the parents/legal guardians of all non-adult participants.

## Author Contributions

ZB and SP conceptualized the research question. RP, DB, AB, and BR assisted with data preparation and analysis. RP drafted the manuscript. All authors provided the critical revisions.

## Conflict of Interest Statement

The authors declare that the research was conducted in the absence of any commercial or financial relationships that could be construed as a potential conflict of interest.
