# Supplementary material for: Abused Children Experience High Anger Exposure
Source: Front Psychol. 2019 Mar 5;10:440. doi: 10.3389/fpsyg.2019.00440 (PMC6411659; doi:10.3389/fpsyg.2019.00440)
Supplement: Supplementary file 1 [file Data_Sheet_1.docx]

Supplemental Materials

**Additional Participant Information**

**Recruitment.** Families were recruited from the community through television and print ads, flyers placed in neighborhood centers, and mailings sent home through public elementary schools. The University Institutional Review Board approved all research. Parents provided written consent for verification of child maltreatment reports involving their child; these reports were held by the county’s Child Protective Services (CPS) agency through the state’s Department of Human Services.

**Exclusions.** An additional 1239 individuals were excluded from the sample because they were either missing a relevant questionnaire score (i.e., the questionnaire was not included in a particular study, the participant chose not to fill out the questionnaire, or the participant did not fill out the questionnaire completely) or because they were duplicate participants (child or sibling participated in more than one study; we included each family only once in this study even if they participated in multiple experiments or had multiple children in our studies). If a parent/guardian had a child or children who participated in more than one study, then the parent’s highest AX-Index score was selected. The logic here is that parents may monitor their self-report in a laboratory environment; therefore, higher anger scores (within an individual) may reflect a less filtered response. If a parent had the same questionnaire score across multiple visits, the earliest date was selected.

**Additional Measure Information**

**Conflict Tactic Scale.** Sample items include: “Burned or scalded him/her on purpose” and “Shook him/her.” Respondents were instructed to estimate how often their child experienced each of 31 events in the past year along an 8-point forced-choice response scale (1 = once in the past year, 2 = twice in the past year, 3 = 3-5 times in the past year, 4 = 6-10 times in the past year, 6 = more than 20 times in the past year, 7 = not in the past year but it happened before, 0 = this has never happened), resulting in scores ranging from 0 to 325.

**STAXI-2.** Spielberger and Reheiser (2004) provide evidence in support of the item homogeneity, reliability, and validity of the STAXI-2. In regard to item homogeneity, based on the STAXI-2 normative sample (N > 1,900), a median Cronbach alpha coefficient of .87 has been reported, with alphas for the AX-Index ranging from .75 to .82. Spielberger (1999) reports high internal consistency, with all alpha coefficients exceeding .72 and a median alpha of *r* = .88 (see S1 for means, standard deviations, and alphas by subscale). Borteyrou, Bruchon-Schweitzer, & Spielberger (2008) report test-retest (stability) coefficients over a two-month interval for a French adaptation of the STAXI-2 (N = 139) of .70 for the Trait Anger scale and .32 for the State Anger scale, respectively (in accord with state-trait theory).

**STAXI-2 Subscales.** The 15-item State Anger scale (S-Ang) measures the extent to which a person feels angry at a particular moment, while the 10-item Trait Anger scale (T-Ang) measures how often angry feelings are experienced over time. The 8-item Anger Expression-In scale (AX-I) assesses how often angry feelings are experienced but not expressed (suppressed), whereas the 8-item Anger Expression-Out scale (AX-O) captures how often angry feelings are expressed in verbally or physically aggressive behavior. The 8-item Anger Control-In scale (AC-I) measures how often a person attempts to control angry feelings by calming down or cooling off, whereas the 8-item Anger Control-Out scale (AC-O) evaluates how often a person controls the outward expression of angry feelings.

*S1.* Descriptive statistics for STAXI-2 based on normative sample, including alphas (from Spielberger, 1999).

| Measure | Description | Mean (SD) | Alpha |
| --- | --- | --- | --- |
| AX_Index | Anger Expression | 32.86 (13.37) | .76 |
| S_Ang | State Anger | 18.58 (6.08) | .93 |
| T_Ang | Trait Anger | 18.15 (5.18) | .85 |
| AX-I | Anger Expression- Inward | 16.12 (4.18) | .76 |
| AX-O | Anger Expression- Outward | 15.06 (3.72) | .74 |
| AC-I | Anger Control- Inward | 22.94 (5.87) | .92 |
| AC-O | Anger Control- Outward | 23.37 (5.06) | .85 |

References

Borteyrou, X., Bruchon-Schweitzer, M., and Spielberger, C. D. (2008). The French

adaptation of the STAXI-2. C.D. Spielberger’s state-trait anger expression

inventory. L’Encephale 34, 249–255. doi: 10.1016/j.encep.2007.06.001

Spielberger, C. D. (1999). Staxi-2: State-trait Anger Expression Inventory-2;

Professional Manual. Odessa, FL: PAR, Psychological Assessment Resources.

Spielberger, C. D., and Reheiser, E. C. (2004). “Measuring anxiety, anger,

depression, and curiosity as emotional states and personality traits with

the STAI, STAXI, and STPI,” in Comprehensive Handbook of Psychological

Assessment: Personality Assessment, Vol. 2, eds M. Hersen, D. L. Segal, and M.

Hilsenroth (New York, NY:Wiley), 74–80.
